# Patterns of Psychiatric Referrals in a Consultation-Liaison Setting at a Rural Tertiary Care Center in Central India

**DOI:** 10.7759/cureus.90431

**Published:** 2025-08-18

**Authors:** Anantprakash S Saraf, Santanu Nath, Harshal S Sathe, Bijit Biswas

**Affiliations:** 1 Psychiatry, Government Medical College, Rajnandgaon, IND; 2 Psychiatry, All India Institute of Medical Sciences, Deoghar, IND; 3 Psychiatry, Mahatma Gandhi Institute of Medical Sciences, Wardha, IND; 4 Community and Family Medicine, All India Institute of Medical Sciences, Deoghar, IND

**Keywords:** adjustment disorder, comorbidity, consultation liaison psychiatry, depression, india, psychiatry referral

## Abstract

Introduction: Consultation-liaison psychiatry (CLP) addresses psychiatric symptoms in patients referred from non-psychiatric departments. This study examined the socio-demographic, clinical, and referral profiles of such patients in a rural tertiary care medical institution in central India.

Materials and methods: All patients referred to the psychiatry department over a two-month period were included. Data on demographics, referral patterns, and diagnoses were collected using a structured proforma and analyzed descriptively with 95% confidence intervals (CI).

Results: Of the 200 patients included in the study, 151 patients (75.5%, 95% CI: 69.1-80.9) were referred from inpatient settings. The general medicine department contributed the majority of referrals, accounting for 162 cases (81.0%, 95% CI: 75.0-85.8). The most frequent medical diagnosis among referred patients was poisoning, reported in 78 cases (39.0%, 95% CI: 32.5-45.9), followed by alcoholic liver disease in 15 cases (7.5%, 95% CI: 4.6-12.0). The most common reasons for psychiatric referral were self-harm behavior in 80 patients (40.0%, 95% CI: 33.4-46.9), multiple unexplained physical symptoms in 25 patients (12.5%, 95% CI: 8.6-17.8), and alcohol withdrawal and de-addiction in 23 patients (11.5%, 95% CI: 7.7-16.7). The leading psychiatric diagnoses included major depressive disorder in 28 patients (14.0%, 95% CI: 9.9-19.5), adjustment disorder in 25 patients (12.5%, 95% CI: 8.6-17.8), acute stress reaction in 25 patients (12.5%, 95% CI: 8.6-17.8), delirium in 20 patients (10.0%, 95% CI: 6.5-14.9), and somatoform disorder in 11 patients (5.5%, 95% CI: 3.1-9.6). Regarding management, 87 patients (43.5%, 95% CI: 36.8-50.4) received pharmacological treatment alone, while 83 patients (41.5%, 95% CI: 34.9-48.4) were treated with both pharmacological and non-pharmacological approaches. Follow-up adherence was noted in 75 patients (37.5%, 95% CI: 31.0-44.4).

Conclusion: Despite significant psychiatric morbidity, referral rates remain low. Strengthening CLP services and improving psychiatric training among non-psychiatric clinicians are essential, especially in rural and resource-constrained settings.

## Introduction

Consultation-liaison psychiatry (CLP) is a subspecialty of psychiatry focused on the evaluation and management of psychiatric symptoms in patients admitted to non-psychiatric units of a general hospital [1]. It facilitates effective collaboration among professionals, enabling expert input regarding diagnosis and management. CLP encompasses clinical, teaching, and research activities conducted by mental health professionals within non-psychiatric divisions of a general hospital [2].

Studies have demonstrated that psychiatric comorbidities are relatively common among medical and surgical patients. The reported prevalence ranges from 27% to 46.5% in international studies, while Indian studies indicate that 10% to 36% of general hospital outpatients present with some form of psychiatric morbidity [3-7]. These figures are notably higher than the prevalence of psychiatric disorders in the general population. The ideal approach to managing such psychiatric manifestations involves appropriate referrals to specialists, a practice central to CLP. This strategy has been shown to reduce the burden of illness on both patients and healthcare systems [8].

Psychiatric comorbidity in medical inpatients is associated with prolonged hospital stays and increased utilization of medical services, which subsequently lead to a higher treatment cost burden [9]. Despite this understanding, referral rates from other clinical specialties to psychiatry remain low [10]. Indian studies have reported inpatient referral rates between 0.1% and 1.4% and outpatient referral rates between 0.1% and 2.4%, with most referrals originating from internal medicine departments rather than surgical units [11,12].

Several factors influence referrals to psychiatry from non-psychiatric units. Chen et al. (2016) reviewed these and categorized them into systemic, referrer-related, and patient-related factors. Systemic factors include the availability of a dedicated CLP service and a designated CLP consultant. Referrer-related factors involve the physician’s knowledge of psychiatry and comfort in making psychiatric referrals. Patient-related factors include younger age, a history of psychiatric illness, and the presence of psychosis [13].

The most common reasons for referrals to psychiatric services are depression, delirium, substance use, and self-harm [11,12,14]. Although emergency psychiatry referrals are under-researched in India, available studies report that the most frequent reasons for psychiatric consultation in emergency departments include suicide attempts, agitation, violence, and altered sensorium [15]. One study noted that a large proportion of inpatient and outpatient psychiatry referrals involved patients with medically unexplained physical symptoms (MUPS), who were later diagnosed with neurotic, stress-related, or somatoform disorders [16]. The diagnostic concordance between referring physicians and psychiatrists remains low, highlighting the importance of effective interdisciplinary communication [15].

While CLP has contributed significantly to integrating psychiatry into general medical care, there remains a dearth of research on this subject in India [2]. Current postgraduate training in psychiatry and psychology appears insufficient in equipping professionals for this crucial subspecialty [13]. To the best of our knowledge, no studies have yet explored CLP practices in rural medical settings. Therefore, the current study was conducted, which aimed to assess the patterns of referrals made to the psychiatry department by other medical specialties in a tertiary care institution located in a rural tertiary healthcare center.

## Materials and methods

Study design and setting

This descriptive, cross-sectional study was conducted over a two-month period, from 1 March to 30 April 2019, in the Department of Psychiatry at Mahatma Gandhi Institute of Medical Sciences, Sevagram, Wardha, Maharashtra, India. The institute primarily caters to a rural population. The study was initiated following approval from the Institutional Ethics Committee of Mahatma Gandhi Institute of Medical Sciences, Wardha, India (Approval No.: MGIMS/IEC/PSY/02/2019).

Participants and recruitment

All patients who were referred to the psychiatry department for consultation during the study period from various clinical departments, including inpatient wards, outpatient services, and the intensive care unit (ICU), were included. A total of 200 such patients were consecutively recruited. Written informed consent was obtained from all participants prior to inclusion.

Data collection procedure

Data were collected using a structured proforma. The proforma captured socio-demographic details (such as age, sex, marital status, education level, occupation, and place of residence), source of referral (inpatient department (IPD), outpatient department (OPD), or ICU), referring department, and clinical information relevant to the reason for psychiatric referral.

Medical diagnoses, as recorded in the referring department, were noted for each patient. The reasons for psychiatric referral were categorized based on the primary clinical concern (e.g., self-harm behavior, MUPS, alcohol withdrawal, abnormal behavior, or disorientation).

All referred patients underwent a psychiatric assessment. Wherever a formal psychiatric diagnosis could be made, it was assigned according to the International Classification of Diseases, Tenth Revision (ICD-10) Classification of Mental and Behavioural Disorders - Diagnostic Criteria for Research [17]. Multiple psychiatric conditions, including depressive disorders, adjustment disorders, stress-related disorders, delirium, substance use disorders, somatoform disorders, schizophrenia, and organic psychiatric conditions, were diagnosed.

Management and follow-up

Each case was assessed regarding the need for pharmacological, non-pharmacological, or combined treatment. Interventions were delivered either exclusively within the referring clinical department or through transfer to the psychiatry ward, when necessary. The nature of management provided - pharmacological, non-pharmacological, both, or none - was documented for all patients. Follow-up status was recorded based on whether the patient returned for a scheduled outpatient review.

Statistical analysis

All collected data were entered into Microsoft Excel (Microsoft® Corp., Redmond, WA, USA) and analyzed using JAMOVI software version 2.6.26 (The JAMOVI Project, Sydney, Australia). Descriptive statistics were used throughout. Categorical variables such as referral source, referring department, reason for referral, psychiatric diagnosis, type of management, and follow-up adherence were presented as frequencies and percentages, along with corresponding 95% confidence intervals (CIs). No inferential statistical tests were applied, and the analysis was limited to descriptive reporting.

## Results

Socio-demographic profile

A total of 200 patients were referred to the psychiatry department during the two-month data collection period. The socio-demographic characteristics of the study population are presented in Table 1. Among the participants, 118 (59.0%) were male, and the remaining were female. The mean age was 36.0 ± 17.3 years. Most participants were married (120; 60%) and had an educational level below the 10th standard (124; 62.0%). A significant majority (144; 72.0%) came from rural areas and belonged to nuclear families (139; 69.5%). In terms of occupation, the largest group comprised farmers (95; 47.5%), followed by students (40; 20.0%) and homemakers (35; 17.5%). A smaller segment of the population (13; 6.5%) was unemployed.

**Table 1:** Socio-Demographic Profile of Referred Patients (N = 200) CI: confidence interval

Socio-Demographic Profile		Frequency	Percentage (%)	95% CI
Sex	Male	118	59.0	52.1-65.6
	Female	82	41.0	34.4-47.9
Marital Status	Married	120	60.0	53.1-66.5
	Unmarried	80	40.0	33.5-46.9
Domicile Family	Rural	144	72.0	65.4-77.7
	Semi-Urban	26	13.0	9.0-18.4
	Urban	30	15.0	10.7-20.6
	Nuclear	139	69.5	62.8-75.5
	Joint	61	30.5	24.5-37.2
Education	Below 10th	124	62.0	55.1-68.4
	10th and Above	76	38.0	31.5-44.8
Occupation	Unemployed	13	6.5	3.8-10.8
	Farmer	95	47.5	40.7-54.4
	Business	4	2.0	0.8-5.0
	Professional	9	4.5	2.4-8.3
	Government	4	2.0	0.8-5.0
	Student	40	20.0	15.0-26.1
	Housewife	35	17.5	12.8-23.4

Sources of referrals

Of the total referrals, 151 (75.5%) were inpatient consultations, where a call was made to the psychiatry department to assess patients admitted in various wards. Additionally, 39 (19.5%) of the cases were referred to the Psychiatry OPD, while 10 (5.0%) referrals originated from the hospital’s ICU. Among the referring specialties, the general medicine department accounted for the majority of cases, contributing 162 (81.0%) of all referrals for consultation-liaison services (Table 2).

**Table 2:** Source, Medical Diagnosis and Reasons for Referral to the Psychiatry Department (N = 200) CI: confidence interval; k/c/o: known case of

Referral Profile		Frequency	Percentage (%)	95% CI
Setting	Inpatient (IPD)	151	75.5	69.1-80.9
	Outpatient (OPD)	39	19.5	14.6-25.5
	Intensive Care Unit (ICU)	10	5.0	2.7-8.9
Referring Departments	General Medicine	162	81	75.0-85.8
	General Surgery	8	4	2.0-7.7
	Paediatrics	7	3.5	1.7-7.0
	ENT	5	2.5	1.1-5.7
	Obstetrics and Gynaecology	4	2.0	0.8-5.0
	Orthopaedics	4	2.0	0.8-5.0
	Neurosurgery	4	2.0	0.8-5.0
	Dermatology	4	2.0	0.8-5.0
	Ophthalmology	1	0.5	0.1-2.8
	Dental	1	0.5	0.1-2.8
Medical Diagnoses	Poisoning	78	39.0	32.5-45.9
	Alcoholic Liver Disease	15	7.5	4.6-12.0
	Diabetes Mellitus	10	5	2.7-8.9
	Epilepsy	9	4.5	2.4-8.3
	Others	88	44.0	37.3-50.9
Reasons for Referral	Self-Harm Behaviour	80	40.0	33.4-46.9
	Multiple Unexplained Physical Symptoms	25	12.5	8.6-17.8
	Alcohol Withdrawal and De-addiction	23	11.5	1.8-16.7
	Confused and Disoriented Patient	19	9.5	6.2-14.4
	Irrelevant Talk/Abnormal Behaviour	19	9.5	6.2-14.4
	Uncooperative Patient	8	4.0	2.0-7.7
	Irritable Mood and Behaviour	7	3.5	1.7-7.0
	Anxious/Fearful Patient	5	2.5	1.1-5.7
	k/c/o Psychiatric Disorder	5	2.5	1.1-5.7
	Aggression/Agitation	3	1.5	0.5-4.3
	Forgetfulness/Cognitive Impairment	2	1.0	0.3-3.6
	Sexual Problems	2	1.0	0.3-3.6
	Sleep Problems	2	1.0	0.3-3.6

Medical diagnosis and reasons for referral

Among the referred patients, the most common medical diagnosis was poisoning, accounting for 78 cases (39.0%), most of whom were admitted under the general medicine department. Other notable diagnoses included alcoholic liver disease (15; 7.5%), diabetes mellitus and its complications (10; 5.0%), epilepsy (9; 4.5%), and trauma-related conditions and others (88; 44.0%). The reasons for psychiatric referral were varied. The most frequent cause was self-harm or suicidal behavior, observed in 80 patients (40.0%). This was followed by MUPS in 25 patients (12.5%) and alcohol withdrawal or de-addiction needs in 23 patients (11.5%). Disorientation or confused behavior (19; 9.5%) and irrelevant speech or abnormal behavior (19; 9.5%) were also common indications. Additional reasons included uncooperative behavior (8; 4.0%), irritable mood (7; 3.5%), anxious or fearful behavior (5; 2.5%), and a known history of psychiatric illness (5; 2.5%). Less common reasons for referral were aggression/agitation (3; 1.5%), forgetfulness or cognitive impairment (2; 1.0%), sleep problems (2; 1.0%), and sexual issues (2; 1.0%) (Table 2).

Psychiatric diagnosis and management

The most common psychiatric diagnosis among referred patients was major depressive disorder, noted in 28 cases (14.0%). Adjustment disorder and acute stress disorder were each diagnosed in 25 patients (12.5%). Delirium was identified in 20 cases (10.0%), while somatoform disorders were diagnosed in 11 patients (5.5%). Alcohol-related disorders were also prevalent: alcohol withdrawal symptoms (AWS) in 15 patients (7.5%), alcohol dependence syndrome (ADS) in six (3.0%), and alcohol intoxication in five (2.5%). Schizophrenia was diagnosed in eight patients (4.0%). Other diagnoses included dissociative disorder (6; 3.0%) and intellectual disability with behavioral issues (5; 2.5%). In five cases (2.5%), no formal psychiatric diagnosis was established (Tables 3-4).

**Table 3:** Psychiatric Diagnosis Given to the Patients Brought Under Consultation-Liaison Services (N = 200) CI: confidence interval; NOS: not otherwise specified

Psychiatric Diagnosis	Frequency	Percentage (%)	95% CI
Major Depressive Disorder	28	14	9.9-19.5
Acute Stress Reaction	25	12.5	8.6-17.8
Adjustment Disorder	25	12.5	8.6-17.8
Delirium	20	10	6.5-14.9
Alcohol Withdrawal Syndrome	15	7.5	4.6-12.0
Somatoform Disorder	11	5.5	3.1-9.6
Schizophrenia	8	4.0	2.0-7.7
Alcohol Dependence	6	3.0	1.4-6.4
Conversion Disorder	6	3.0	1.4-6.4
Alcohol Intoxication	5	2.5	1.1-5.7
Intellectual Disability With Behavioural	5	2.5	1.1-5.7
No Psychiatric Disorder	5	2.5	1.1-5.7
Organic Mental Disorder	4	2.0	0.8-5.0
Alcohol Harmful Use	3	1.5	0.5-4.3
Alcohol-Induced Psychosis	3	1.5	0.5-4.3
Hypochondriasis	3	1.5	0.5-4.3
Personality Disorder	3	1.5	0.5-4.3
Alzheimer's Dementia	2	1.0	0.3-3.6
Vascular Dementia	2	1.0	0.3-3.6
Delirium Tremens	2	1.0	0.3-3.6
Acute Transient Psychotic Disorder	2	1.0	0.3-3.6
Psychosis NOS	2	1.0	0.3-3.6
Bipolar Affective Disorder	2	1.0	0.3-3.6
Panic Disorder	2	1.0	0.3-3.6
Erectile Dysfunction	2	1.0	0.3-3.6
Others	5	2.5	1.1-5.7

**Table 4:** Managements Offered to the Consultation Liaison Psychiatry Cases (N = 200) CI: confidence interval

Consultation-Liaison Profile		Frequency	Percentage (%)	95% CI
Management	No management	1	0.5	0.1-2.8
	Pharmacological	87	43.5	36.8-50.4
	Non-pharmacological	29	14.5	10.3-20.0
	Both	83	41.5	34.9-48.4
Transfer to the Psychiatry Ward	Yes	17	8.5	5.4-13.2
	No	183	91.5	86.8-94.6
Follow-Up	Yes	75	37.5	31.0-44.4
	No	125	62.5	55.6-68.9

## Discussion

In this descriptive, cross-sectional study at a rural tertiary care centre in Maharashtra, all consecutive patients referred to psychiatry over two months were assessed using a structured proforma. Nearly three in five were men, about two-thirds had education below the secondary level, and nearly three-fourths lived in rural areas, most in nuclear families. Farming was the main occupation for almost one in two patients. Three in every four referrals came from inpatient services, with over four in five from general medicine. Poisoning affected about two in five patients, and self-harm was the leading reason for referral. Depression, stress-related, and adjustment disorders each affected around one in eight patients. Management often combined medicines with counselling, and fewer than one in ten required admission to the psychiatry ward.

A substantial proportion (72.0%) of patients were from rural backgrounds, which is consistent with the location of the institute. Most patients belonged to nuclear families (69.5%) and were predominantly farmers (47.5%). These findings are in line with those of Hashim et al. (2018), who reported that 90.4% of patients referred for suicidal risk originated from rural areas, and 49.5% were engaged in farming [18].

In our study, the majority (75.5%) of referrals were received from inpatient wards, while 19.5% were from the Psychiatry OPD, and 5% originated from the ICU. This distribution differs from other Indian studies, where outpatient referrals accounted for a larger share (59.0-74.8%) and inpatient referrals ranged from 41.0% to 69.7% [10,12,16]. This contrast may be attributed to the rural nature of the setting, where the absence of super-specialty departments often results in higher IPD admissions in general medicine, thereby increasing the volume of inpatient psychiatry referrals.

The general medicine department accounted for 81.0% of all referrals, surpassing other specialties. This is likely due to the limited availability of medical super-specialties (except neurology) at this rural medical college, leading to patients requiring advanced care being admitted under general medicine. This trend of general medicine being the predominant source of CLP referrals is supported by both national and international literature [12,16,19]. Surgical departments collectively accounted for 13.5% of referrals in our study - a figure that aligns with findings from studies in Northeast India (11.8-17.8%) [12,20], but falls slightly short compared to South Indian studies [10,16]. These differences could be due to variations in how individual studies classify "surgical branches."

With respect to the reasons for consultation, deliberate self-harm (DSH) and suicidal behavior were the most common causes (40.0%) in our study. Previous Indian studies have reported similar referral rates for suicidal behavior, ranging from 5.73% to 51.4% [12,16,18,20,21]. MUPS accounted for 12.5% of referrals, making it the second most common reason. This is consistent with earlier studies, where MUPS referrals ranged from 2.5% to 47.5% [10,12,16,20]. Disorientation, irrelevant speech, and abnormal behavior collectively accounted for 19% of referrals - similar to previously reported figures ranging from 13.1% to 70.2% [10,20,21].

As for substance use disorders, only one case of alcohol dependence with AWS (11.5%) was recorded. The low prevalence of other substance-related referrals may be due to their culturally sanctioned use in rural communities, which might deter patients from seeking treatment. Nevertheless, the referral rate for alcohol use disorders in our setting falls within the range observed in urban Indian studies (5.7-18.4%) [10,12,20,21].

In terms of psychiatric diagnoses, the most common was major depressive disorder (14.0%). This aligns with previous Indian studies reporting prevalence between 7.9% and 19.2%, and with broader affective disorder diagnoses constituting around 8.0% of all CLP cases [10,18,20,21]. However, the proportion of affective disorders in studies from outside India, such as Nepal (27.0%) and Europe (18.7%), is relatively higher [22,23]. Adjustment disorder and acute stress disorder were each diagnosed in 12.5% of cases, aligning with reported ranges from 7.64% to 42.5% in Indian studies [16,18,20].

Our study reported a lower prevalence of delirium (10%) compared to other Indian studies, which have reported rates between 17.8% and 21.6% [20,21]. This may reflect limited awareness among clinicians regarding the psychiatrist’s role in managing delirium, despite its classification as an organic psychiatric disorder. Somatoform disorders were also less frequently diagnosed (5.5%) compared to one South Indian study reporting 23.0% [10].

Schizophrenia was diagnosed in 4% of the referred cases, comparable to European findings [23], but higher than those reported from Nepal and parts of India [20,22]. Dissociative disorders were identified in only 3% of patients - lower than the 6.3% to 18.7% reported in studies conducted in more urban settings [16,20,21]. This discrepancy may stem from stronger cultural explanations for dissociative symptoms in rural populations, which could hinder identification within a clinical model.

Regarding management, pharmacotherapy alone was the most commonly adopted approach (43.5%), followed by a combination of pharmacological and non-pharmacological interventions (41.5%). This trend aligns with the findings of Tekkalaki et al. [12] and Bhogale et al. [16]. In high-volume OPD settings, exclusive delivery of psychotherapy or counseling may not always be feasible due to time constraints. Furthermore, patients may be more accepting of pharmacological treatment than psychotherapeutic modalities unless referred explicitly to a psychiatric setting [12]. Nonetheless, we emphasize that an individualized and integrative approach - combining both pharmacological and psychological strategies - is essential for optimal care in CLP.

This study has certain limitations. First, the duration of data collection was short, and the sample size was limited. Second, the rural context of our institution limits the generalizability of findings to other settings. Third, the absence of super-specialty departments prevented us from examining referral patterns from such units. Fourth, a longitudinal design might have offered better insights into evolving referral trends, compared to the cross-sectional approach used here. Lastly, the study did not assess diagnostic concordance between referring departments and the psychiatry team.

## Conclusions

The present study evaluated CLP services in a rural tertiary care medical institution - likely one of the first studies of its kind in such a setting. The findings revealed that most referred patients were managed within their respective parent departments, without the need for transfer to a psychiatric ward. This highlights the feasibility and effectiveness of integrated psychiatric care through interdepartmental collaboration, which not only facilitates timely intervention but may also help reduce the stigma often associated with psychiatric referrals. Given the unique rural context and the absence of super-specialty services, the study provides valuable insights into how CLP functions in resource-constrained settings. Future research should focus on longitudinal assessments to capture evolving referral patterns, clinical outcomes, and diagnostic concordance. Such evidence would further strengthen the knowledge base of CLP and guide its structured implementation in similar settings across the country.
